# Radiolabeling and Preliminary In Vivo Evaluation of the Candidate CCR2 Targeting PET Radioligand [^11^C]AZD2423

**DOI:** 10.3390/ph18020135

**Published:** 2025-01-21

**Authors:** Kenneth Dahl, Peter Johnström, Miklós Tóth, Martin Bolin, Katarina Varnäs, Ryuji Nakao, Akihiro Takano, Yasir Khani Meynaq, Malken Bayrakdarian, Zsolt Cselényi, Christer Halldin, Lars Farde, Magnus Schou

**Affiliations:** 1 PET Science Centre, Precision Medicine and Biosamples, Oncology R&D, AstraZeneca, Karolinska Institutet, 171 76 Stockholm, Sweden; peter.johnstrom@astrazeneca.com (P.J.); zsolt.cselenyi@astrazeneca.com (Z.C.); magnus.schou@astrazeneca.com (M.S.); 2 Department of Clinical Neuroscience, Centre for Psychiatry Research, Karolinska Institutet and Stockholm County Council, 171 76 Stockholm, Sweden; miklos.toth@ki.se (M.T.); martin.bolin@ki.se (M.B.); katarina.varnas@ki.se (K.V.); ryuji.nakao@ki.se (R.N.); akihiro.takano@ki.se (A.T.); yasir.khani@ki.se (Y.K.M.); christer.halldin@ki.se (C.H.); lars.farde@ki.se (L.F.); 3 AstraZeneca NS IMED, Montreal, QC H4S 1Z9, Canada; malken.bayrakdarian@astrazeneca.com; 4 HUN-REN TKI, Department of Biophysics and Radiation Biology, Semmelweis University, 1094 Budapest, Hungary

**Keywords:** carbonylation, radiochemistry, radiopharmaceutical, Chemokine Receptor 2, PET imaging

## Abstract

Background: AZD2423 is a high-affinity and selective negative allosteric modulator of the chemokine receptor type 2 (CCR2). This receptor plays important roles in the extravasation and transmigration of monocytes under inflammatory conditions. The aims of the current positron emission tomography (PET) study were as follows: (i) to develop an efficient synthetic method for labeling AZD2423 with carbon-11 (^11^C, t_1/2_ = 20.4 min) and (ii) to evaluate its potential to visualize CCR2 binding in the non-human primate (NHP) brain. Methods: [^11^C]AZD2423 was synthesized using a novel two-step, two-pot [^11^C]carbon monoxide carbonylation procedure. PET imaging studies in NHPs (n = 2) were conducted to assess its brain penetration and in vivo distribution. Results: Radiolabeling of [^11^C]AZD2423 was accomplished with good yield (7.4 ± 0.6%, n = 4) and high radiochemical purity (>99%) using [^11^C]carbon monoxide. Preliminary PET imaging in NHPs revealed low [^11^C]AZD2423 brain exposure under both baseline and pretreatment conditions (SUV_peak_ = 0.4, n = 2). However, high concentrations of radioactivity were observed in organs outside the brain at baseline, e.g., the thyroid gland (SUV_peak_ = 3.3, n = 2), parotid gland (SUV_peak_ = 3.4, n = 2), and submandibular gland (SUV_peak_ = 4.4, n = 2). This radioactivity was markedly reduced following pretreatment with AZD2423 (3.0 mg/kg), indicating specific binding of [^11^C]AZD2423 to CCR2 in vivo. The presence of specific CCR2 binding was further validated using two-tissue compartment modeling, which demonstrated a 59–63% reduction in the total volume of distribution values in the analyzed peripheral tissues. Conclusions: Altogether, [^11^C]AZD2423 shows potential as a PET radioligand for the in vivo visualization of CCR2 expression in tissues outside the brain and may also serve as a lead compound for the further development of a CCR2 PET radioligand suitable for brain imaging.

## 1. Introduction

The Chemokine Receptor 2 (CCR2) is a G protein-coupled receptor pivotal in regulating immune cell migration and trafficking [1,2,3]. Within the central nervous system (CNS), CCR2 is primarily expressed in monocytes and microglia, which are crucial components of the brain’s immune defense. Consequently, this receptor has been studied in various conditions characterized by inflammation within the CNS, such as multiple sclerosis, Alzheimer’s disease, and ischemic stroke [1,4,5,6]. Given CCR2’s involvement in various inflammatory and immune-related diseases, the development of imaging techniques to visualize CCR2 expression in vivo has generated significant interest in both preclinical and clinical research.

Positron Emission Tomography (PET) imaging utilizes radiolabeled ligands (so-called radioligands) to target specific biomolecules, allowing for the visualization and quantification of their distribution and expression in various tissues and organs in vivo [7,8,9]. PET imaging of radioligand binding to CCR2 holds promise for advancing our understanding of immune responses in various pathological conditions and may have implications for the development and evaluation of targeted therapies [10].

So far, only a handful of radioligands have been applied in PET imaging of the CCR2 receptor. The most noteworthy is the peptidic ligand [^64^Cu]DOTA-ECL1i that binds to the first extracellular loop of the CCR2 receptor ECL1i [11,12]. This ligand has, for example, been successfully applied to visualize an increased CCR2 expression in a rat model of abdominal aortic aneurysm (AAA), a leading cause of death in the aging population [13]. Further to this, its gallium-68 labeled analog [^68^Ga]DOTA-ECL1i has shown promise as a non-invasive imaging biomarker for cardiovascular disease [14].

**Figure 1:**
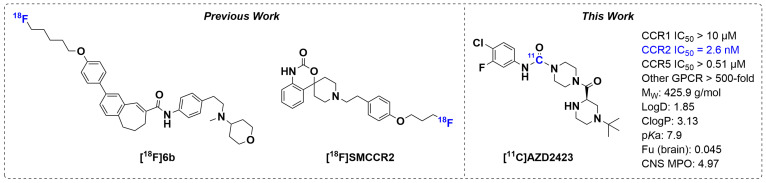
Chemical structures of novel small molecule CCR2 PET ligands. Previously reported radioligands [^18^F]6a and [^18^F]SMCCR2 (**Left**) and [^11^C]AZD2423 (**Right**).

The pursuit to develop a small-molecule PET radioligand that targets CCR2 to access the CNS has thus far been less successful. To date, there are two compounds presented in the literature, [^18^F]6b and [^18^F]SMCCR2 (Figure 1) [15,16]. [^18^F]6b demonstrated good in vitro affinity (14 nM) and selectivity to CCR2; however, its in vivo stability and specificity for CCR2 have yet to be confirmed. On the other hand, [^18^F]SMCCR2 has been evaluated in vivo, but its low affinity for CCR2 (3–6 µM) will restrict its utility as a PET radioligand. Hence, there is still a need for more sensitive and specific radioligands to assess CCR2 expression in vivo and, in particular, within the CNS.

AZD2423 is a negative allosteric modulator of the CCR2 receptor, originally developed for the treatment of neuropathic pain [17,18,19]. AZD2423 has many of the desired properties for a successful PET ligand intended for neuroimaging (Figure 1), which include, high affinity (2.6 nM) and good selectivity for CCR2, a molecular weight < 500 g/mol, moderate Log D (1.85), as well as favorable CNS multiparameter optimization (MPO, 4.97) [20] and brain slice distribution (0.045) values.

The aim of the current study was two-fold: (i) to develop an efficient synthetic method for labeling AZD2423 with carbon-11 (^11^C, t_1/2_ = 20.4 min) and (ii) to evaluate its brain exposure and regional brain distribution in non-human primates (NHPs) with PET. This is a step towards the development of a PET radioligand for in vivo visualization of CCR2 binding in the human brain.

## 2. Results and Discussion

### 2.1. Radiolabeling of [^11^C]AZD2423 and Automation

Because AZD2423 lacks a terminal methyl group, the standard ^11^C-methylation protocol was not pursued. Instead, a high throughput labeling approach relying on [^11^C]carbon monoxide ([^11^C]CO) was adopted [21,22]. [^11^C]CO, generated from in-target produced [^11^C]carbon dioxide, is a particularly appealing synthon in PET radiochemistry due to the prevalence of the carbonyl group in biologically relevant molecules. Various ^11^C-labeled functional groups have been labeled with [^11^C]CO, for example, [^11^C]amides, [^11^C]esters, [^11^C]carboxylic acids, and [^11^C]ureas [23]. The latter, [^11^C]urea, is of specific interest here as AZD2423 contains an aromatic urea. The Rh-mediated ^11^C-carbonylation of aromatic azides to prepare [^11^C]ureas was first described by Doi et. al. [24] and this protocol has successfully been applied in our laboratory [25].

**Figure 2:**
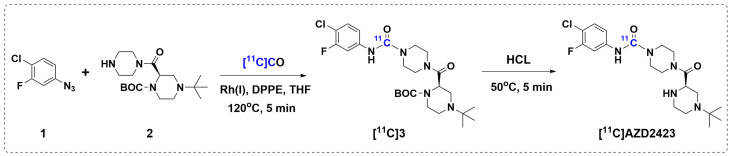
Radiosynthesis of [^11^C]AZD2423 via a two-step and two-pot [^11^C]CO carbonylation procedure.

Based on this background, a novel two-pot and two-step carbonylation reaction was devised, aiming to label AZD2423 with ^11^C in the urea position (Figure 2). In brief, [^11^C]CO was first reacted with an aromatic azide (**1**, Figure 2) and secondary amine precursor (**2**, Figure 2) in the presence of a Rh-catalyst using a high-pressure reactor system. The resulting mixture was further treated with aqueous hydrochloric acid in a second vessel to furnish the final product. As expected, using the condition outlined in Figure 2, [^11^C]AZD2423 was obtained as the major product.

Carbon-11 labeled radioligands for in vivo PET imaging studies are typically produced in giga-becquerel quantities. As our goal was to develop a synthesis methodology suitable for routine radioligand production, the [^11^C]AZD2423 synthesis process was fully automated to improve its reliability and reproducibility. The complete process was performed using a previously described [^11^C]CO prototype synthesis apparatus (GEMS, Uppsala, Sweden) [25], which included the initial formation of the crude [^11^C]AZD2423 product, followed by its isolation by semipreparative high-performance liquid chromatography (HPLC), solid-phase extraction (SPE), and sterile filtration to yield the formulated product. Using these conditions, [^11^C]AZD2423 was prepared with radioactivities exceeding 1 GBq and the radiochemical purity (RCP) was greater than 99%. The synthesis process took approximately 40 min, and the molar activity (Am) at the time of injection ranged from 15 to 20 GBq/μmol (405 to 540 Ci/mmol). Although the Am achieved in this study was slightly lower than that of other radioligands produced through ^11^C-carbonylation, it was comparable to other radioligands synthesized using [^11^C]CO in our laboratory. The average radiochemical yield for the synthesis of [^11^C]AZD2423 was 7.4 ± 0.6% (n = 4, relative to the [^11^C]CO_2_ at the start of synthesis). The identity of the labeled compound was verified by co-injecting it with a reference standard. Additionally, the radioligand [^11^C]AZD2423 demonstrated stability in a formulation of 9% ethanol in PBS (phosphate-buffered saline, pH 7.4) for up to 60 min.

### 2.2. PET Imaging Studies in Non-Human Primates

To evaluate the brain exposure and regional distribution of [^11^C]AZD2423 in non-human primate (NHP) brains, PET imaging studies were carried out. Thus, two 123 min dynamic brain PET measurements were performed at baseline and after pretreatment with unlabeled AZD2423 (3 mg/kg), respectively.

Average PET images (3–123 min, Figure 3) and time-activity curves (TACs) were generated for the two conditions (Figure 4). As evident from the PET images (Figure 3), [^11^C]AZD2423 displayed low brain exposure at both baseline and pretreatment conditions (peak standardized uptake value (SUV_peak_) = 0.4, n = 2, Figure 4A). CNS MPO has emerged as a valuable tool for predicting the blood-brain barrier (BBB) permeability of new drugs and a score value ≥ 4.0 is generally desirable [20]. The low brain exposure of [^11^C]AZD2423 was thus unexpected when keeping in mind the rather high CNS MPO score of 4.97. A potential explanation for the observed poor brain permeability is that [^11^C]AZD2423 is a substrate for a transporter protein at the BBB (e.g., PgP).

**Figure 4:**
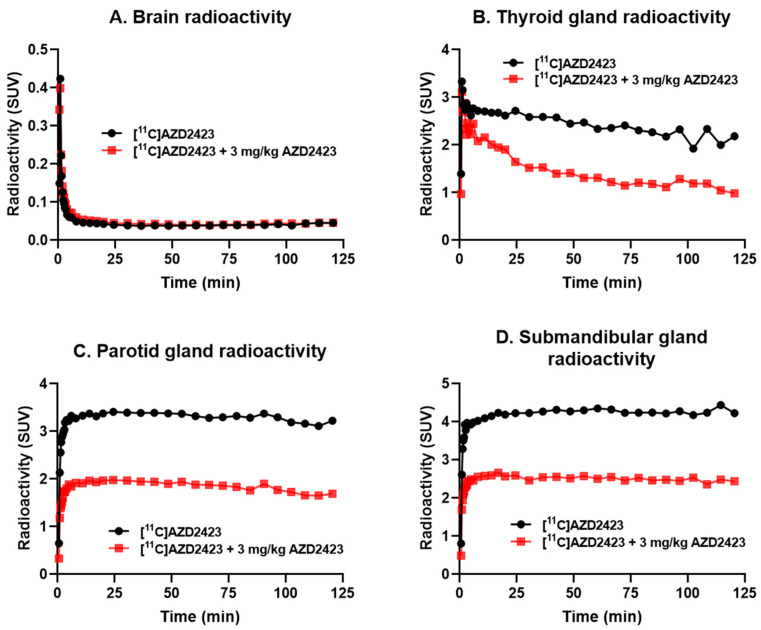
Average time-activity curves (n = 2) for the brain (**A**), thyroid gland (**B**), parotid gland (**C**), and submandibular gland (**D**) at baseline (filled circles) and pretreatment conditions (filled boxes) following intravenous administration of [^11^C]AZD2423.

The field of view included the head and neck. High radioactivity concentrations were, on the other hand, observed in organs outside the brain at baseline, including the thyroid (SUV_peak_ = 3.3, n = 2, Figure 4B), parotid (SUV_peak_ = 3.4, n = 2, Figure 4C), and submandibular glands (SUV_peak_ = 4.4, n = 2, Figure 4D). This radioactivity concentration was markedly reduced following pretreatment with AZD2423 (3 mg/kg). The percentage reduction in the total volume of distribution (V_T,_ two-tissue compartment analysis) values relative to baseline was between 59 and 63% in all investigated peripheral tissues (Table 1). Similar results (49–51% decrease) were also obtained when evaluating the area under the curve for the respective regions (data shown in the Supporting Information). Thus, the quantitative analyses provided support for the hypothesis that a substantial proportion of [^11^C]AZD2423 binding was specific to the CCR2 receptor in vivo. This interpretation is consistent with the high affinity and high unbound fraction of [^11^C]AZD2423 demonstrated in tissue in vitro [26].

A limitation of the current study is that the pretreatment study was conducted after administration of the same ligand as that used in imaging (e.g., autologous competition). Therefore, although unlikely, it cannot be excluded based on the exquisite in vitro selectivity of AZD2423 that the reduction in binding in part represents off-target binding. Future research involving [^11^C]AZD2423 will concentrate on examining its whole body distribution under both healthy and inflammatory conditions. Additionally, medicinal chemistry efforts to modify AZD2423 are underway to improve its ability to cross the blood-brain barrier while preserving its high affinity for CCR2.

### 2.3. Radiometabolite Analysis

**Figure 5:**
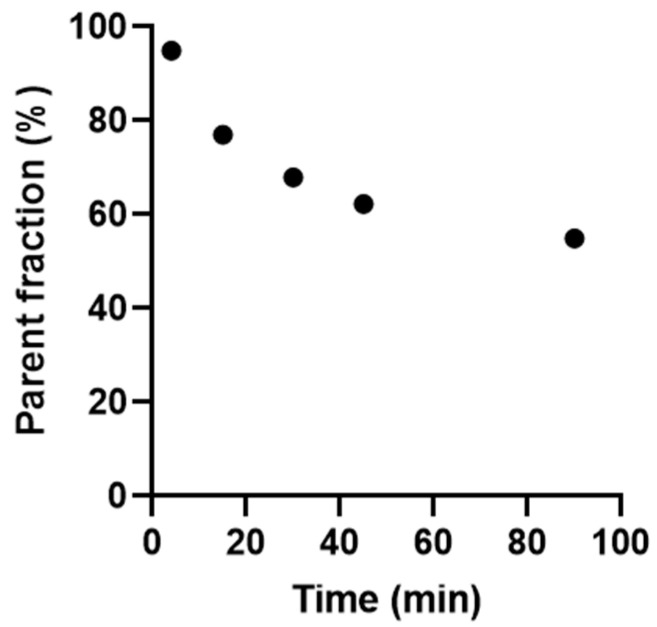
Time-course for parent radioligand in plasma following the intravenous administration of [^11^C]AZD2423.

To measure parent radioligand and radiometabolites in plasma, discrete arterial blood samples were drawn at five time points after the injection of [^11^C]AZD2423. The radioligand was metabolized rather slowly (Figure 5). At 10 min after injection, approximately 80% of the radioactivity in plasma represented parent radioligand, decreasing to approximately 60% at 90 min. Most radiometabolites detected in plasma eluted with a retention time (t_R_) of 2.1–2.7 min and were thus less lipophilic than [^11^C]AZD2423 (t_R_ = 3.0 min). However, one metabolite with a longer retention time (t_R_ = 3.5 min) was formed (see Supporting Information). These metabolites were not identified and further work would be required to fully characterize these unknown radiometabolites. However, such work was outside the scope of this study.

## 3. Materials and Methods

Unless specified otherwise, all reagents were sourced from commercial suppliers and used without additional purification. The precursors **1** and **2**, as well as the reference standard for AZD2423 were provided by AstraZeneca (Figure 1).

### 3.1. Radiochemistry

No-carrier-added [^11^C]CO_2_ (~50 GBq) was generated by irradiating a nitrogen and oxygen gas mixture (containing 0.5% oxygen) with 16.4 MeV protons following the ^14^N(p,α)^11^C nuclear reaction. [^11^C]CO_2_ was converted to [^11^C]CO using heated molybdenum (850 °C) and subsequently reacted with the coupling reagents (chlorofluorophenyl azide (**1**, 10 mg), amine precursor (**2**, 5 mg), [Rh(cod)Cl]dimer (3.5 mg), and 1,2-Bis(diphenylphosphino)ethane (dppe, 0.4 mg)) in anhydrous THF (700 µL) at 120 °C for 5 min using a high-pressure microautoclave system as previously described elsewhere [25]. Following the ^11^C-carbonylation procedure, the resulting solution was heated (50 °C) with HCl (6M (aq), 250 µL) for another 5 min to yield the crude [^11^C]AZD2423 product. Purification and formulation were conducted using a computer-controlled automated system from DMAutomation, Sweden. Semipreparative HPLC was carried out with a reversed-phase C-18 column (μBondapak, 10 μm, 10 × 300 mm, Waters) using an eluent of MeCN-HCO_2_NH_4_ (10 mM) in a 35:65 *v*/*v* ratio at a flow rate of 6 mL/min. The column’s outlet was connected to an absorbance detector set at a wavelength of 254 nm and in series with a radiation detector. The purified product, with a retention time of 12 min, was diluted in 50 mL of water and subjected to further purification using solid phase extraction (SepPak, tC18 plus short, Waters). The product was formulated in 9% ethanol in PBS (phosphate-buffered saline, pH 7.4) and finally passed through a sterile filter (0.22 μm sterile Millex-GV filter, Merck KGaA, Darmstadt, Germany) to generate a sterile product that is ready to use in preclinical PET studies. Analytical chromatograms and methods for [^11^C]AZD2423 can be found in the Supporting Information.

### 3.2. PET Imaging in Non-Human Primates

PET experiments were conducted on two anesthetized healthy rhesus monkeys weighing 5.3 and 4.7 kg. The study received approval from the Stockholm Animal Research Ethical Committee (Dnr. N452/11) and is comprehensively described in Dahl et al. [27]. Anesthesia was induced via an intramuscular injection of ketamine hydrochloride (approximately 10 mg/kg, Ketalar, Pfizer AB, Stockholm, Sweden) and maintained with a mixture of sevoflurane (2–8%, Abbott Scandinavia AB, Solna, Sweden), oxygen (around 40%), and medical air following endotracheal intubation. The anesthesia level was continuously monitored during the experiment. The head was stabilized using a fixation device, and the body temperature was regulated with a Bair Hugger model 505 (Arizant Healthcare, St. Paul, MN, USA), with continuous monitoring via an esophageal thermometer. ECG, heart rate, blood pressure, respiratory rate, and oxygen saturation were all continuously monitored throughout the experiments.

Two PET measurements were performed on the same experimental day in each of the two monkeys. Baseline PET measurements were conducted using [^11^C]AZD2423 with doses of 164 MBq and 157 MBq. A pretreatment PET measurement was also performed, during which an infusion of unlabeled AZD2423 (3.0 mg/kg) occurred over 10 min, starting 30 min before the injection of [^11^C]AZD2423 (90 MBq and 158 MBq). In each PET experiment, the radioligand was administered as an intravenous bolus injection over 5 s, coinciding with the start of PET data acquisition. Radioactivity in the brain was continuously measured for 123 min using the high-resolution research tomograph (HRRT; Siemens Molecular Imaging, Knoxville, TN, USA), following a preprogrammed sequence of 34 frames. Arterial blood samples were collected at specified time points (4, 15, 30, 45, and 90 min) and analyzed for radioactivity in blood and plasma, as well as for the presence of the remaining parent compound, [^11^C]AZD2423.

### 3.3. Kinetic Model Analysis

The Regions of Interest (ROIs) were delineated manually on MRI images of each NHP for the whole brain, thyroid gland, parotid gland, and submandibular gland. The summed PET images of the entire duration were co-registered with the MRI image of the individual NHP. After applying the co-registration parameters to the dynamic PET data, time–activity curves were generated for all ROIs for each PET measurement. The average SUV was calculated for each ROI. Target occupancy was estimated using the volume of distribution (V_T_) calculated by a two-tissue compartment model (2TC) with metabolite-corrected plasma radioactivity.

## 4. Conclusions

Radiolabeling of [^11^C]AZD2423 was accomplished in good yield (7.4 ± 0.6%, n = 4) and at high radiochemical purity (>99%) using [^11^C]CO. PET studies in NHPs revealed limited brain exposure of [^11^C]AZD2423 at both baseline and pretreatment conditions (SUV_peak_ = 0.4). The limited brain exposure of [^11^C]AZD2423 may be attributed to its potential as a substrate for a transporter protein at the BBB. Interestingly, at baseline, high levels of radioactivity (SUV_peak_ = 3.3–4.4) were observed in peripheral organs, including the thyroid gland, parotid gland, and submandibular gland. This radioactivity was also reduced after pretreatment with AZD2423, indicating specific binding of [^11^C]AZD2423 to CCR2 in vivo. The presence of CCR2 specific binding was further validated using two-tissue compartment modeling, which demonstrated a 59–63% reduction in V_T_ values in the analyzed peripheral tissues. Altogether, [^11^C]AZD2423 shows potential as a PET imaging biomarker for in vivo visualization of CCR2 expression of organs outside the brain. [^11^C]AZD2423 may also serve as a lead compound for further development of a CCR2 PET radioligand suitable for brain imaging.

## Figures and Tables

**Figure 3:**
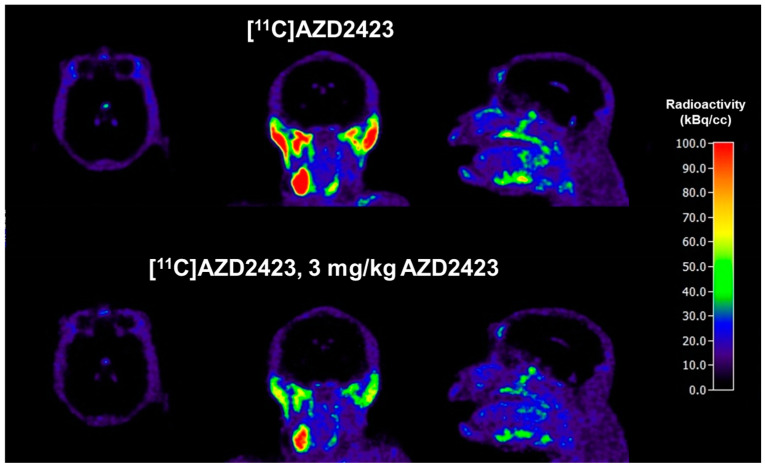
Color-coded PET images showing distribution of radioactivity in the monkey head and neck after radioligand injection at baseline and after pretreatment with unlabeled AZD2423. Summation images from 3 to 123 min are shown. Image intensity normalized for injected radioactivity.

**Table 1:** Average V_T_ values (n = 2, two-tissue compartment analysis) at baseline and pretreatment conditions, and percentage decrease.

Periphel Organ	Baseline	Pretreatment	Percentage Decrease (%)
Thyroid gland	53.1	19.4	63.4
Parotid gland	111.1	41.6	62.6

## Data Availability

The data generated and analyzed during this research will be shared by the corresponding author upon reasonable request.

## Supplementary Materials

The following supporting information can be downloaded at: https://www.mdpi.com/article/10.3390/ph18020135/s1.
