# Supplementary material for: Radiolabeling and Preliminary In Vivo Evaluation of the Candidate CCR2 Targeting PET Radioligand [11C]AZD2423
Source: Pharmaceuticals (Basel). 2025 Jan 21;18(2):135. doi: 10.3390/ph18020135 (PMC11858205; doi:10.3390/ph18020135)
Supplement: Supplementary file 1 [file pharmaceuticals-18-00135-s001.zip › pharmaceuticals-3384926-supplementary.pdf]

# Radiolabeling and preliminary in vivo evaluation of the candidate CCR2 targeting PET radioligand [$^{11}$C]AZD2423

Kenneth Dahl [1,2]*, Peter Johnström[1,2], Miklós Tóth[2], Martin Bolin[2], Katarina Varnäs[2], Ryuji Nakao[2], Akihiro Takano[2], Yasir Khani Meynaq[2], Malken Bayrakdarian[3], Zsolt Cselényi[1,2], Christer Halldin[2], Lars Farde[2] and Magnus Schou[1,2]

[1]PET Science Centre, Precision Medicine and Biosamples, Oncology R&D, AstraZeneca, Karolinska Institutet, 171 76 Stockholm, Sweden

[2]Department of Clinical Neuroscience, Centre for Psychiatry Research, Karolinska Institutet and Stockholm County Council, 171 76 Stockholm, Sweden

[3]AstraZeneca NS IMED, Montreal, QC H4S 1Z9, Canada

*Correspondence: kenneth.dahl@astrazeneca.com

# Supporting Information

**Content**

1) Analytical methods and HPLC chromatograms for [$^{11}$C]AZD2423.
2) Percentage reduction data for [$^{11}$C]AZD2423 binding relative to baseline following the pretreatment of AZD2423 (3.0 mg/kg).
3) Method description and representative radioactivity HPLC trace for radiometabolite analysis.

### *Analytical methods and HPLC chromatograms*

Analytical HPLC method used for quality control (QC).
**Column:** C-18 Zorbax eclipse (5 µm, 150 x 4.6 mm, Agilent)
**Mobile phase:** MeCN / 0.1 M HCO2NH4
**Injection volume:** 5 µL
**Method:** Gradient, 10% MeCN for 1 min, 10% MeCN to 90% in 5 min, 90% for 2 min (Run time = 7 min, flow rate = 3 mL/min, λ = 254 nm)

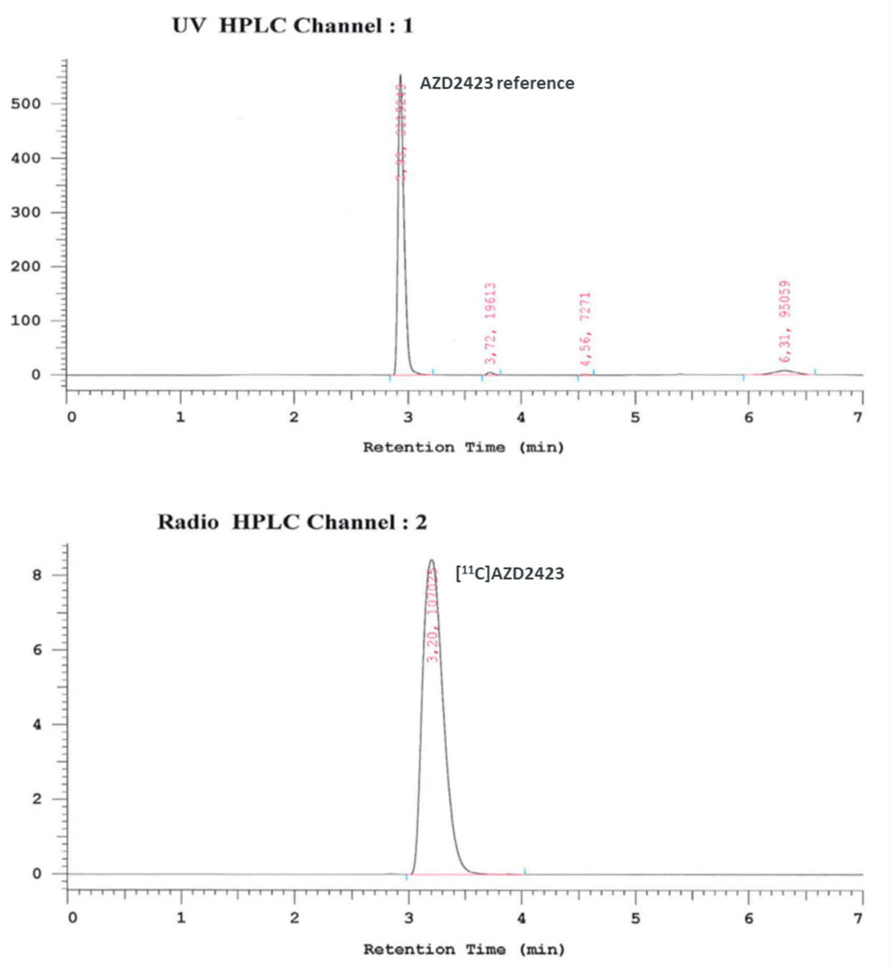


**Figure S1.** Representative HPLC chromatogram for [$^{11}$C]AZD2423 and co-injection with unlabeled reference standard. UV trace (top) and Radioactivity trace (bottom).

***Percentage reduction data (averege of n = 2 NHP experiments) for [$^{11}$C]AZD2423 binding relative to baseline following the pretreatment of AZD2423 (3.0 mg/kg).***

| | AUC (Thyroid) | AUC (Blood) | AUC (Thyroid)/AUC (Blood) | Procentage reduction (%) |
|---|---|---|---|---|
| Baseline | 290 | 36 | 8.1 | 49.4 |
| Pretreatment | 173 | 42 | 4.1 | |

**Table S1.** Percentage reduction data for [$^{11}$C]AZD2423 binding in the Thyroid gland relative to baseline.

| | AUC (Parotid) | AUC (Blood) | AUC (Parotid)/AUC (Blood) | Procentage reduction (%) |
|---|---|---|---|---|
| Baseline | 395 | 36 | 11 | 51.2 |
| Pretreatment | 222 | 42 | 5.3 | |

**Table S2.** Percentage reduction data for [$^{11}$C]AZD2423 binding in the Parotid gland relative to baseline.

| | AUC (Submandibular) | AUC (Blood) | AUC (Submandibular)/AUC (Blood) | Procentage reduction (%) |
|---|---|---|---|---|
| Baseline | 509 | 36 | 14.1 | 49 |
| Pretreatment | 302 | 42 | 7.2 | |

**Table S3.** Percentage reduction data for [$^{11}$C]AZD2423 binding in the Submandibular gland relative to baseline.

***Method description and representative radioactivity HPLC trace for radiometabolite analysis.***

After collection, the blood was centrifuged at 4000 rpm for 2 minutes to separate the plasma. The plasma was then diluted with 1.4 times its volume of acetonitrile and centrifuged at 6000 rpm for 4 minutes. The resulting extract was separated from the pellet and further diluted with water (3 mL). HPLC for radiometabolite analysis was performed using a radio-HPLC system consisted of an interface module (D-7000; Hitachi: Tokyo, Japan), a L-7100 pump (Hitachi), an injector (model 7125, with a 5.0-mL loop; Rheodyne: Cotati, USA), and an ultraviolet absorption detector (L-7400, 254 nm; Hitachi) in series with a 150TR; Packard radioactivity detector (housed in a shield of 50 mm thick lead) equipped with a 550 µL flow cell. HPLC method are described below.

HPLC method used for metabolite analysis.
**Column:** C-18 XBridge (5 µm, 100 x 10 mm, Waters)
**Mobile phase:** MeCN / 20 mM ammonium phosphate (pH 7)
**Injection volume:** 5 mL
**Method:** Gradient, 1% MeCN for 1 min, 1% MeCN to 50% in 4 min (Run time = 5 min, flow rate = 6 mL/min, λ = 254 nm)

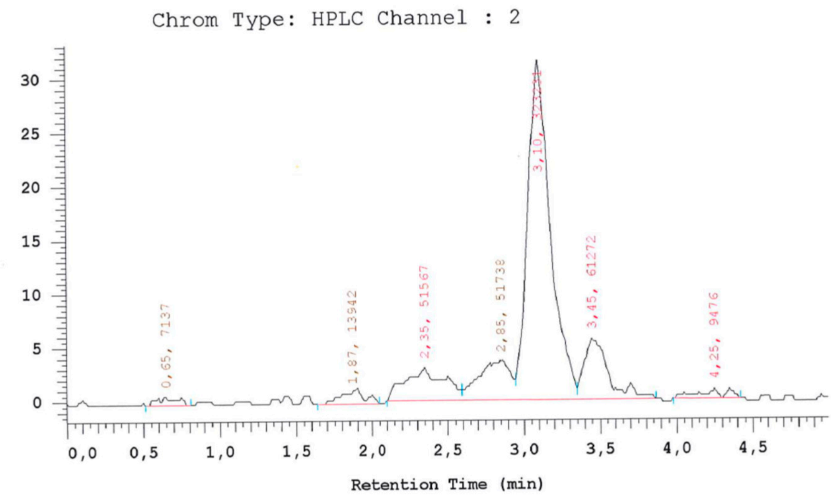


**Figure S2.** Representative HPLC chromatogram for the radiometabolite analysis of [$^{11}$C]AZD2423 (Rt = 3.1 min). The figure shows the radioactivity signal for the 30 minutes sample following iv administration of [$^{11}$C]AZD2423.
